# Adult-Onset Alexander Disease Presenting as Atypical Parkinsonism and Autonomic Dysfunction: A Case Series

**DOI:** 10.3390/jcm15145677

**Published:** 2026-07-20

**Authors:** Jinping Fang, Zhan Wang, Tao Feng, Ying Jiang

**Affiliations:** 1 Parkinson Medical Center, Beijing Rehabilitation Hospital, Capital Medical University, Beijing 100144, China; 2 Center for Movement Disorders, Department of Neurology, Beijing Tiantan Hospital, Capital Medical University, Beijing 100070, China; 3 China National Clinical Research Center for Neurological Diseases, Beijing Tiantan Hospital, Capital Medical University, Beijing 100070, China

**Keywords:** adult-onset Alexander disease, GFAP, parkinsonism, case report, tadpole sign

## Abstract

**Introduction**: Adult-onset Alexander disease (AOAD) is a rare astrocytopathy linked to the glial fibrillary acidic protein (GFAP) gene, which is known for its clinical heterogeneity and common misdiagnosis. In adults, it may present with bulbar dysfunction, pyramidal signs, ataxia, dysautonomia, cognitive decline, or parkinsonism, which often mimics atypical parkinsonian syndromes like multiple system atrophy (MSA). The purpose of this case series was to define practical clinical, radiological, and genetic cues for suspecting AOAD in adults with atypical parkinsonism, autonomic dysfunction, or paroxysmal focal symptoms, particularly when genetic findings are inconclusive. **Case Presentation**: Four patients, aged 40 to 59 years, had progressive and varied neurological symptoms, like gait disturbance, lower limb weakness, dysarthria, dysphagia, autonomic dysfunction, parkinsonism, cognitive decline, and paroxysmal focal deficits. Initial diagnoses were parkinsonian syndrome, stroke, transient ischemic attack (TIA), and MSA. **Diagnostic Assessment and Intervention**: Brain magnetic resonance imaging (MRI) in all patients showed characteristic lower brainstem abnormalities, particularly atrophy of the medulla oblongata and upper cervical spinal cord, consistent with the “tadpole sign.” GFAP sequencing identified one likely pathogenic variant (p.Arg70Trp) and three variants of uncertain significance: p.Glu122_Arg124del, p.Met415Ile, and p.Glu195Val. Management was mainly symptomatic; one patient showed significant motor improvement after repetitive transcranial magnetic stimulation (rTMS). **Conclusions**: AOAD should be considered in adults with parkinsonism-plus syndromes or unexplained combinations of bulbar symptoms, pyramidal signs, autonomic dysfunction, and cognitive decline. Recognizing the tadpole sign on MRI may improve diagnostic accuracy, particularly when genetic results are inconclusive.

## 1. Introduction

Alexander disease (AxD; OMIM: 203450) is a rare, autosomal dominantly inherited neurological disorder caused by mutations in the glial fibrillary acidic protein (*GFAP*; OMIM: 137780) leading to abnormal expression of this protein and accumulation inside astrocytes as Rosenthal fibres [1,2]. Current evidence supports a toxic gain-of-function mechanism rather than simple loss of GFAP function: pathogenic GFAP variants disturb intermediate-filament assembly, reduce GFAP solubility, and promote accumulation of mutant or overexpressed GFAP together with ubiquitin and small heat-shock proteins. These changes trigger proteo-static stress, reactive astrogliosis, and inflammatory signaling, thereby contributing to white-matter injury and selective vulnerability of the brainstem and spinal cord [3,4,5]. GFAP-lowering strategies and modulation of astrocytic stress pathways, including STAT3 and lipocalin-2, are therefore being explored as potential dis-ease-modifying directions [6,7]. Clinically, it shows variable features depending on the patient’s age and is classified into three groups namely infantile, juvenile and adult-onset forms, although the latter is very rare [8]. Childhood-onset AxD is generally divided into infantile onset at 0–2 years and juvenile onset at 3–12 years, while adult-onset disease is usually defined as onset after 13 years of age; neonatal and other early-childhood presentations have also been reported, supporting a continuum rather than a rigid age boundary [8,9]. The classic clinical features for diagnosing adult-onset Alexander disease (AOAD) are dysarthria, dysphagia, and/or bulbar dysfunction (i.e., difficulty in speaking or eating), as well as pyramidal signs (spastic paresis) and ataxia of the cerebellar origin. However, autonomic dysfunction, cognitive impairment, and sleep disturbances are common, contributing to its heterogeneous and variable clinical phenotype [10]. In adults, symptomatology is also highly variable and not specific, often leading to diagnostic confusion with other neurodegenerative diseases [11]. For example, adult AxD can present in a clinical manner that is similar to Parkinson’s disease, multiple system atrophy (MSA) or several forms of spinocerebellar ataxias, or in some cases, amyotrophic lateral sclerosis [12,13,14,15].

Imaging is an important part of diagnosis. Imaging features are often present in adults presenting with AxD including a marked reduction in size of the medulla and upper spinal cord at cervical C1–C2 levels (tadpole sign) and/or a high T2 signal in the medullary and dentate nuclei of the cerebellum as well as a variable degree of involvement of periventricular or fronto-parietal white matter. In a few instances, contrast-enhancement may be observed within the meninges or brain stem tissue and recognizing of these unique radiologic features often leads to targeted genetic testing [16]. Many GFAP mutations are associated with AxD, which has been described in the literature with a total of 182 mutations reported to date [9]; it is inherited in an autosomal dominant manner. Recent genetic data suggest that AxD is underdiagnosed, with prevalence estimates up to 1.42 per 100,000, far above traditional estimates [17]. Previously described adult-associated pathogenic or disease-causing GFAP variants and their reported adult phenotypes are summarized in Table 1.

Here, we report four adult cases of AOAD with atypical and diagnostically misleading presentations, including parkinsonism, autonomic dysfunction, and paroxysmal focal neurological episodes. The purpose of this publication is to define practical clinicoradiological–genetic clues that should prompt suspicion of AOAD in adults, rather than simply reiterate previously known features of the disease. By integrating clinical phenotype, characteristic MRI findings, three-generation pedigrees, and GFAP variant interpretation, this series aims to show how AOAD can be recognized even when genetic findings are classified as variants of uncertain significance.

## 2. Case Descriptions

### 2.1. Case 1

A 58-year-old woman presented with a 5-year history of progressive gait disturbance and lower-limb weakness. Her symptoms worsened during the preceding 6 months. She had previously been diagnosed with a parkinsonian syndrome. The illness began with bilateral leg weakness and recurrent falls, and she later developed gait instability and eventually required assistance to walk. Her key associated features were dysarthria, hypophonic speech, dysphagia with choking on liquids, constipation, urinary incontinence, episodic dizziness, rapid eye movement (REM) sleep-related abnormal behaviors, anxiety, depression, and recent short-term memory decline. A three-generation family history was obtained, and no family history of similar neurological symptoms or known neurodegenerative disease was reported. Neurological examination showed dysarthria, mild cognitive impairment, left-gaze nystagmus, and left-sided cerebellar impairment. Her gait was slow and wide-based. The pull test was positive. Deep tendon reflexes were increased in the left lower limb. The left plantar response was extensor. Brain magnetic resonance imaging (MRI) showed marked atrophy of the medulla oblongata and thinning of the upper cervical spinal cord, as well as supratentorial white-matter changes (Figure 1a–d). Autonomic testing showed reduced sympathetic skin response amplitudes, indicating sympathetic dysfunction. Whole-exome sequencing identified a heterozygous GFAP in-frame deletion, c.363_371del (p.Glu122_Arg124del), classified as a variant of uncertain significance. Genetic testing was performed at the Beijing Tiantan Hospital–Shenzhen BGI Medical Genetics Laboratory, Shenzhen, China, and peripheral-blood genomic DNA was analyzed by single-proband capture-based high-throughput whole-exome sequencing. This identified a heterozygous GFAP in-frame deletion, NM_002055.5:c.363_371del (p.Glu122_Arg124del), located in exon 1 and classified as a variant of uncertain significance according to ACMG criteria, with supporting evidence PM2 and PM4. No Sanger confirmation or trio-based analysis was documented in the available report. The corresponding three-generation pedigree is shown in Figure 2A.

### 2.2. Case 2

A 40-year-old man presented with a 2-year history of progressive left-sided weakness and dysarthria. He was initially diagnosed with parkinsonian syndrome, and later, cerebrovascular disease was also suspected. The illness began with left-sided limb weakness and slurred speech, with no tremor or sensory disturbance. One year later, his symptoms worsened after a minor fall. He then developed left-sided rigidity, bradykinesia, reduced arm swing, dragging of the left leg, and dysphagia with choking on liquids. Levodopa treatment (125 mg three times daily) produced no clinical benefit. Neurological examination showed dysarthria, left-sided pyramidal signs, left-sided extrapyramidal signs, mild cerebellar incoordination, brisk tendon reflexes, and bilateral Hoffmann signs. Cognitive function was preserved (Mini-Mental State Examination [MMSE] 30/30; Montreal Cognitive Assessment [MoCA] 29/30). Autonomic symptoms were absent. Brain MRI showed marked atrophy of the brainstem and medulla oblongata, with mild frontal white-matter abnormalities (Figure 1e). Orthostatic blood pressure testing was normal. A three-generation pedigree was obtained, with a positive family history: the patient’s maternal grandfather, mother, and maternal aunt reportedly had similar neurological symptoms, suggesting possible autosomal dominant inheritance through the maternal line. His father was unaffected, and his two offspring had not been clinically or genetically confirmed at the time of evaluation. Genetic testing was performed at the Beijing Tiantan Hospital–Shenzhen BGI Medical Genetics Laboratory, Shenzhen, China, and peripheral-blood genomic DNA was analyzed by single-proband capture-based high-throughput clinical exome sequencing. Genetic testing identified a heterozygous GFAP variant, NM_002055.4:c.1245G>A (p.Met415Ile), located in exon 8 and classified as a variant of uncertain significance according to ACMG criteria, with supporting evidence PM2, PP1, and PP4. Sanger sequencing confirmed the heterozygous variant in the proband. The variant was not detected in the father but was detected in the affected maternal aunt. Complete trio-based exome sequencing was not performed. The corresponding three-generation pedigree is shown in Figure 2B.

### 2.3. Case 3

A 59-year-old woman presented with a 6-year history of progressive parkinsonism, mainly affecting the left side. Her initial symptoms were lower-limb rigidity and bradykinesia. Gait instability developed after 3 years, and she later required a walking aid. She was initially suspected to have multiple system atrophy because of prominent autonomic symptoms. During the disease course, she developed dysarthria, hypophonic speech, dysphagia with choking on liquids, urinary urgency, urinary incontinence, severe nocturia, constipation, and anxiety-depressive symptoms. REM sleep-related dream enactment behavior had been present for more than 10 years. Over the previous 4 years, she also developed progressive short-term memory impairment. She denied hallucinations and hyposmia. A three-generation family history was obtained, with a positive family history: her mother and maternal aunt reportedly had similar neurological symptoms. She had one daughter, who was asymptomatic and genetically untested at the time of evaluation. Neurological examination showed dysarthria, mild cognitive impairment, horizontal nystagmus on left gaze, cerebellar ataxia, and a slow short-stepped gait with bilateral leg dragging. The pull test was positive. Orthostatic hypotension was documented on postural blood pressure testing. Brain MRI showed marked atrophy of the medulla oblongata and upper cervical spinal cord. T2 hyperintensity was present in the bilateral cerebellar dentate nuclei and basal ganglia (Figure 1f). Cognitive assessment showed MMSE 24/30 and MoCA 18/30. Genetic testing identified a heterozygous GFAP variant, c.208C>T (p.Arg70Trp), classified as likely pathogenic. Details of the testing institution, DNA diagnostic method, Sanger confirmation, and trio-based analysis were not documented in the available clinical records. The corresponding three-generation pedigree is shown in Figure 2C.

### 2.4. Case 4

A 47-year-old woman presented with a 1-year history of paroxysmal focal neurological episodes, including limb weakness and numbness. Each episode ran for 10 to 30 min. There was no impairment in awareness, convulsive movements, automatisms, and postictal confusion. She was initially thought to have a transient ischemic attack or another focal neurological disorder. She had these episodic symptoms, like frequent choking on liquids, constipation, mild memory decline, and severe insomnia. She slept for 2 to 3 h each night. After taking oxcarbazepine, her limb numbness improved, but she personally experienced more frequent falls. After reducing the dose, these falls became less frequent. She is currently taking oxcarbazepine 0.15 g twice a day. Neurological examination results show that all four limbs have normal muscle strength and tone, but the tandem gait was impaired, and Romberg sign was very positive. Electroencephalography (EEG) examinations were repeated, and the results were normal. Brain MRI results show that the medulla oblongata exhibits characteristic thinning, and there are abnormal periventricular white-matter hyperintensities (Figure 1g). Genetic testing was performed by Jiaxing AccBio Medical Laboratory, Jiaxing, China, using target-region capture followed by high-throughput sequencing of peripheral-blood genomic DNA. The available report identified a heterozygous GFAP variant, NM_002055.5:c.584A>T (p.Glu195Val), located in exon 3 and classified as a variant of uncertain significance according to ACMG criteria. No Sanger confirmation or trio-based analysis was documented in the available report. The corresponding three-generation pedigree is shown in Figure 2D.

## 3. Discussion

To enhance the relevance of this report, the Discussion now focuses on the specific contribution of our cases rather than on background information alone. This series adds three clinically useful observations: first, AOAD may present as parkinsonism-plus, multiple system atrophy-like disease, stroke/transient ischemic attack-like episodes, or mixed autonomic-bulbar syndromes; second, characteristic cervicomedullary atrophy can guide diagnosis when GFAP variants remain of uncertain significance; and third, family history, pedigree information, and available segregation data are essential for interpreting adult GFAP variants.

AxD is a GFAP-related astrocytopathy. Unlike infantile AxD, adult-onset AxD typically presents with bulbar dysfunction, pyramidal signs, cerebellar ataxia, and autonomic involvement, while cognition may be relatively preserved [3,14]. None of our patients showed palatal myoclonus, highlighting the phenotypic heterogeneity of AOAD [11,15]. Brief summaries of each case are provided below in Table 2; the corresponding three-generation pedigrees are shown in Figure 2; and the genetic testing site, DNA diagnostic method, and available validation/segregation findings are integrated into the case descriptions and Table 2. The four patients in this series illustrate the variability of adult presentations, ranging from a spastic–ataxic gait disorder with autonomic dysfunction (Case 1), to an asymmetric pyramidal–extrapyramidal syndrome (Case 2), to MSA-like parkinsonism with severe dysautonomia (Case 3), and paroxysmal focal neurological episodes (Case 4). Cases 1 and 3 initially resembled MSA, which aligns with previous reports indicating that autonomic dysfunction and reduced striatal dopamine transporter uptake may occur in AOAD [12,13,18]. Cases 2 and 4 further suggest that AOAD may also miss vascular or structural disorders when there are focal symptoms that are asymmetric or episodic. In adults presenting with MSA-like symptoms, such as parkinsonian-plus, or unclear bulbospinal symptoms, especially when there is observed atrophy in the medullary and upper cervical cord, MRI is a vital tool in diagnosing AOAD [13,14,19,20].

For each individual case, it was observed that the pons typically shows relatively preserved tissue, and there was a feature commonly referred to as the ‘tadpole sign’, which is a sign of the pons. This specific MRI pattern is still a very valuable imaging indicator for identifying AOAD, even when genetic findings are not yet definite [16,19]. Other MRI anomalies vary among the enrolled cases, which include medullary and cervical cord signal variations, dentate nucleus involvement, irregularities in the basal ganglia, and supratentorial white matter lesions [21,22]. The collected data indicate a partial but not rigid connection between clinical manifestations and radiological appearances. Shared cervicomedullary atrophy might be the underlying cause of the common bulbospinal manifestations in patients, like speech difficulties, swallowing issues, walking instability, pyramidal symptoms, and autonomic system involvement. The differences in parkinsonism, autonomic dysfunction, cognitive function, and paroxysmal symptoms suggest that the same imaging characteristic does not correspond to a single clinical condition [14,21,22,23]. Quantitative morphometric MRI may further support diagnosis. Proposed midsagittal measurements include a sagittal medulla oblongata diameter of 9.0 mm, a medulla-to-midbrain ratio of 0.60, or a medulla-to-pons ratio of 0.46 [22,24].

Four AOAD patients exhibited distinct GFAP mutations, indicating a significant genetic heterogeneity in adult-onset Alexander disease compared to the clustered mutational hotspots observed in pediatric-onset AxD [23]. In Case 3, the c.208C>T (p. Arg70Trp) variant at the GFAP head–rod junction was linked to asymmetric parkinsonism and severe autonomic dysfunction, which aligns with prior findings and supports the pathogenicity of the variant [12,13]. In Case 2, the c.1245G>A (p.Met415Ile) variant is classified as a variant of uncertain significance (VUS), but its pathogenicity is supported by the characteristic brainstem-predominant MRI changes on MRI, which is a characteristic of the disease, and it has a higher frequency in adult-onset than infantile AxD [9,21]. This distribution of adult-onset variants is biologically plausible, since GFAP filament homeostasis relies on coordinated interactions among the head, rod, and tail domains, and even non-hotspot variants might disrupt filament assembly, reduce solubility, and promote aggregation [25]. In experimental settings, it was found that mutant GFAP accumulates in non-soluble components, forms aggregates with ubiquitin and small heat-shock proteins, and activates stress-response mechanisms, all of which contribute to astrocytic impairment [4,5,26,27]. Variant-specific functional studies were not available for the GFAP variants identified in our cases; therefore, their precise molecular effects cannot be directly inferred. More generally, pathogenic GFAP variants may disrupt intermediate-filament assembly, reduce GFAP solubility, and promote protein aggregation, thereby contributing to astrocytic dysfunction [4,5,27,28]. The resulting disruption of the astrocytic intermediate-filament network, together with GFAP aggregation and cellular stress, may impair astrocytic homeostasis and contribute to progressive neural tissue injury [3,4,5,26,27]. In AOAD, this pathological process predominantly involves the medulla oblongata and upper cervical spinal cord, producing the characteristic cervicomedullary atrophy with relative pontine preservation [14,29]. However, the precise biological basis for this regional vulnerability remains incompletely understood. Regarding molecular testing, the optimal strategy depends on the level of pretest suspicion. In cases where clinical and MRI manifestations strongly suggest AOAD, a focused examination of GFAP gene sequences is a reasonable initial approach [11]. When the clinical presentation is more extensive or has features like hereditary ataxia, leukodystrophy, hereditary spastic paraplegia, or atypical parkinsonism, a multigene panel with GFAP testing might be a better alternative [30]. When the clinical picture is not typical, and when targeted genetic testing gives no positive results, or when adult-onset ataxia is not initially considered, then whole-exome or genome sequencing could be very valuable [11]. Although some GFAP variants have been reported more frequently in adult-onset cases, genotype–phenotype correlations in AOAD remain incompletely understood. Therefore, genetic findings should be carefully interpreted in the context of MRI findings, clinical phenotype, family history, and segregation data when available [15]. This is particularly important for variants of uncertain significance, as shown in Cases 1, 2, and 4.

At the moment, treatment of AOAD is mostly symptomatic; this was the case for the patients examined in our study. However, a few emerging disease-modifying strategies are worth attention. Antisense oligonucleotides targeting GFAP have been shown to clear Rosenthal fibers and reverse pathological changes in preclinical models [6]. Additionally, upstream regulators of astrocytic stress and inflammation, such as STAT3 and lipocalin-2, are being explored as potential therapeutic targets [7]. Moreover, inflammatory features reported in certain adult cases suggest that a portion of patients might benefit from immunomodulatory therapies [31]. Symptom-focused interventions are still vital. Repetitive transcranial magnetic stimulation (rTMS) was applied to three patients (Cases 1–3) during their hospital stay, whereas the outpatient (Case 4) did not receive this treatment. Among them, only Case 3 showed a notable improvement in mobility. During her hospitalization, she had five sessions of rTMS delivered at 5 Hz, which were delivered to the bilateral primary motor cortices (M1) and cerebellar regions. These results suggest that non-invasive neuromodulation might be a helpful additional treatment for AOAD patients with gait issues and parkinsonian-related disabilities. While there is a lack of direct proof specifically in AxD, research on MSA and Parkinson’s disease shows that rTMS provides temporary improvements in motor capabilities and walking ability [32,33,34].

After six months of follow-up, both Cases 1 and 3 showed further deterioration in their walking and balancing abilities, indicating deteriorating motor function over time. Meanwhile, Case 2 developed exertional dyspnea with preserved oxygen saturation, indicating potential involvement of the central or bulbar regions. On the contrary, Case 4 experienced paroxysmal episodes without significant progression. Such observations suggest that AOAD might have a variety of short-term courses, ranging from the gradual deterioration of motor functions to more stable or intermittent symptoms. Progressive gait impairment and bulbar involvement could suggest a more severe functional prognosis, but a longer-term follow-up is necessary.

This study has some limitations; for example, the sample size was small, there was no polysomnographic evaluation, and the analysis was mostly descriptive, so we cannot make any definite clinicoradiological correlations. In addition, complete trio-based exome sequencing and Sanger confirmation were not available for every case; therefore, variants of uncertain significance were interpreted conservatively and in combination with the clinical phenotype, MRI features, family history, and available segregation information. Another limitation is that UPDRS or MDS-UPDRS scores were not available, which prevented standardized quantitative comparison of parkinsonism severity across cases.

## 4. Conclusions

Adult-onset Alexander disease should be considered in adults with atypical parkinsonism, autonomic dysfunction, bulbar or pyramidal signs, and characteristic cervicomedullary atrophy on MRI. The main value of this case series is that it provides a practical diagnostic framework showing that clinical phenotype, MRI, family history, and GFAP genetic findings should be interpreted together, especially when genetic variants are inconclusive.

Reporting guideline: This case series was prepared and reported in accordance with the CAse REport (CARE) guidelines and the CARE extension for case series checklist [35,36].

## Figures and Tables

**Figure 1:**
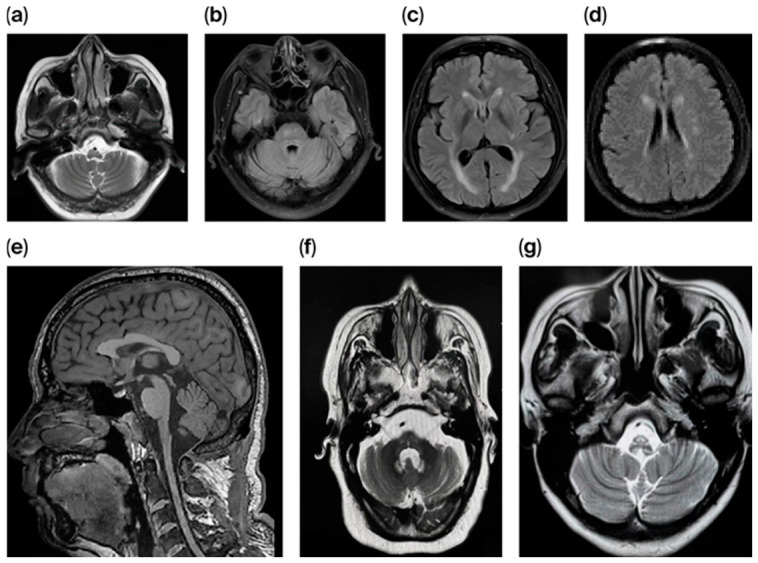
Brain MRI findings across Cases 1–4. (**a**–**d**) Case 1: Brain MRI shows a strong brainstem and supratentorial white matter involvement, with clear atrophy of the medulla oblongata and upper cervical spinal cord. (**e**) Case 2: Sagittal T2-weighted image shows the classic “tadpole sign,” characterized by marked atrophy of the medulla oblongata and upper cervical cord with relative pontine preservation. (**f**) Case 3: Axial T2-weighted image reveals symmetric hyperintensity of the bilateral cerebellar dentate nuclei. (**g**) Case 4: Brain MRI demonstrates characteristic thinning (atrophy) of the medulla oblongata with abnormal signal intensity.

**Figure 2:**
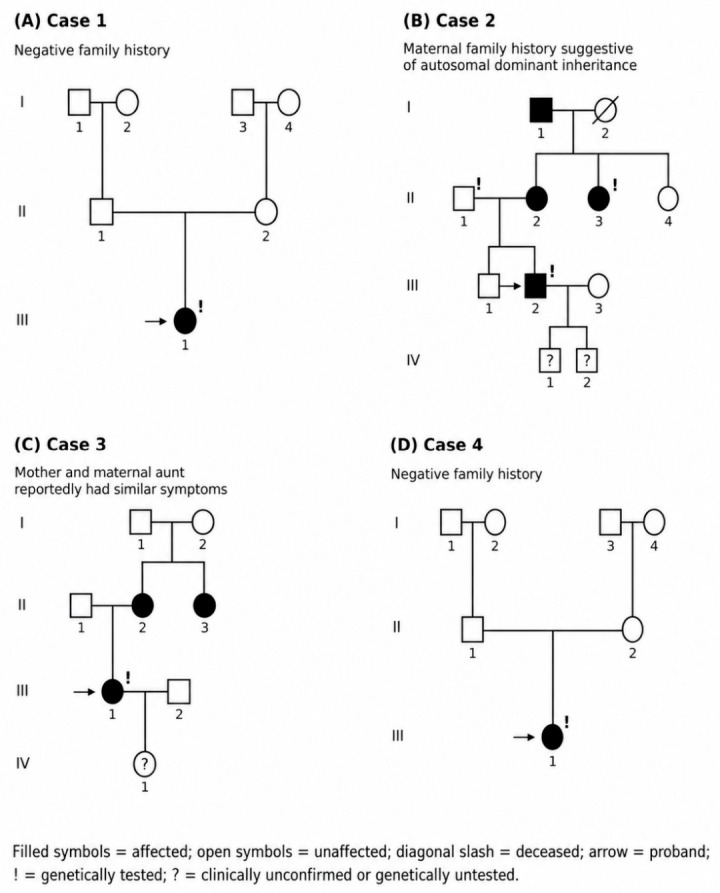
Pedigrees of Cases 1–4 (at least 3 generations). Filled symbols indicate affected individuals; open symbols indicate unaffected individuals; slashed symbols indicate deceased individuals; the arrow indicates the proband; exclamation marks indicate individuals who underwent genetic testing; question marks indicate clinically unconfirmed or genetically untested offspring/relatives.

**Table 1:** Previously described GFAP variants in adult-onset Alexander disease and their reported phenotypic associations.

GFAP Variant	Reported Adult Phenotype(s)	Comment	Key References
p.Arg416Trp (R416W)	Palatal tremor/palatal myoclonus, bulbar dysfunction, pyramidal and cerebellar signs, adult leukodystrophy phenotype	Best-described adult-associated variant; not fully phenotype-specific	[9,10]
p.Asp78Asn (D78N)	Familial adult-onset cerebellar/pseudobulbar syndrome	Supports variable expressivity in familial AOAD	[9]
p.Met74Thr (M74T)	Adult bulbospinal syndrome with dysarthria, dysphagia, spastic gait/tetraparesis, sometimes sensory symptoms or mild cognitive change	Illustrates myelopathic/bulbar adult presentation	[9,10]
p.Tyr257Cys (Y257C)	Progressive ataxia and palatal tremor	Case-based adult association	[9,10]
p.Met73Ile (M73I)	Dysarthria, gait ataxia, spastic tetraparesis	Representative bulbospinal/cerebellar adult phenotype	[9]
p.Leu58Pro (L58P)	Slowly progressive bulbospinal/ataxic phenotype	Newer adult causal variant; limited evidence	[9]
p.Arg70Trp (R70W)	Bulbar, cerebellar, autonomic, or parkinsonian features	Adult-associated pathogenic variant, not phenotype-defining	[9,14]
In-frame deletion variants	Mixed bulbar, pyramidal, and cerebellar manifestations	Phenotypic expansion rather than fixed association	[9]

AOAD, adult-onset Alexander disease; GFAP, glial fibrillary acidic protein. Note: Phenotypic associations are reported adult features and should not be interpreted as deterministic genotype–phenotype correlations.

**Table 2:** Clinical, radiological, and genetic characteristics of patients with adult-onset Alexander disease.

Feature	Case 1	Case 2	Case 3	Case 4
Age (years)/Sex	58/Female	40/Male	59/Female	47/Female
Disease Duration	5 years	2 years	6 years	1 year
Primary Clinical Manifestations	Progressive lower limb weakness, gait instability (“walking on cotton”), urinary incontinence, dysarthria, and dizziness.	Left-sided limb weakness, dysarthria, limb rigidity, and dysphagia.	Parkinsonism (rigidity, bradykinesia), severe autonomic failure (OH, urinary incontinence), and cognitive decline.	Paroxysmal focal weakness and numbness (10–30 min episodes), choking, and severe insomnia.
Cognitive Status (MoCA)	20/30 (mild impairment)	29/30 (preserved)	18/30 (significant impairment)	Mild subjective decline
Family History/Pedigree	Negative; three-generation family history obtained (Figure 2A).	Positive; three-generation maternal family history suggestive of autosomal dominant inheritance (Figure 2B).	Positive; mother and maternal aunt reportedly had similar symptoms; one daughter asymptomatic and genetically untested (Figure 2C).	Negative; three-generation family history obtained (Figure 2D).
Initial Misdiagnosis	Parkinsonism	Stroke/Hemiparesis	Multiple system atrophy (MSA)	Transient ischemic attack (TIA)/focal deficit
Key MRI Findings	Medullary and upper cervical spinal cord atrophy (“Tadpole sign”).	Brainstem and medullary atrophy.	“Tadpole sign”; T2 hyperintensity in cerebellar dentate nuclei and basal ganglia.	Medullary thinning (“Tadpole sign”) and periventricular white matter signals.
GFAP Variant (HGVSc)	c.363_371del	c.1245G>A	c.208C>T	c.584A>T
GFAP Variant (HGVSp)	p.Glu122_Arg124del	p.Met415Ile	p.Arg70Trp	p.Glu195Val
Pathogenicity (ACMG)	VUS	VUS (co-segregation noted)	Likely pathogenic	VUS
Genetic testing site/DNA diagnostic method	Beijing Tiantan Hospital–Shenzhen BGI Medical Genetics Laboratory, Shenzhen, China; single-proband capture-based high-throughput whole-exome sequencing using peripheral-blood DNA.	Beijing Tiantan Hospital–Shenzhen BGI Medical Genetics Laboratory, Shenzhen, China; single-proband capture-based high-throughput clinical exome sequencing using peripheral-blood DNA.	Not documented in the available clinical records.	Jiaxing AccBio Medical Laboratory, Jiaxing, China; target-region capture followed by high-throughput sequencing using peripheral-blood DNA.
Validation/segregation information	No Sanger confirmation or trio-based analysis was documented in the available report.	Sanger sequencing confirmed the heterozygous variant in the proband; the variant was not detected in his father and was detected in the affected maternal aunt. Complete trio-based exome sequencing was not performed.	Sanger confirmation and trio-based analysis were not documented in the available clinical records.	No Sanger confirmation or trio-based analysis documented in the available report.

OH: orthostatic hypotension; MoCA: Montreal Cognitive Assessment; VUS: variant of uncertain significance; ACMG: American College of Medical Genetics and Genomics; MRI: magnetic resonance imaging; HGVSc: Human Genome Variation Society coding DNA sequence nomenclature; HGVSp: Human Genome Variation Society protein sequence nomenclature.

## Data Availability

The datasets presented in this article are not readily available because of ethical and privacy restrictions. Requests to access the datasets should be directed to the corresponding author.
